# New lineage of scuticociliates dominates the ciliate community and bacterivory in hypolimnetic waters of a freshwater reservoir

**DOI:** 10.1093/ismejo/wraf148

**Published:** 2025-07-13

**Authors:** Karel Šimek, Usman Asghar, Bettina Sonntag, Vojtěch Kasalický, Tanja Shabarova, Indranil Mukherjee

**Affiliations:** Biology Centre CAS, Institute of Hydrobiology, Na Sádkách 7, České Budějovice 37005, Czech Republic; Biology Centre CAS, Institute of Hydrobiology, Na Sádkách 7, České Budějovice 37005, Czech Republic; Faculty of Science, University of South Bohemia, České Budějovice 37005, Czech Republic; Research Department for Limnology, Mondsee, University of Innsbruck, Mondseestrasse 9, Mondsee A-5310, Austria; Biology Centre CAS, Institute of Hydrobiology, Na Sádkách 7, České Budějovice 37005, Czech Republic; Biology Centre CAS, Institute of Hydrobiology, Na Sádkách 7, České Budějovice 37005, Czech Republic; Biology Centre CAS, Institute of Hydrobiology, Na Sádkách 7, České Budějovice 37005, Czech Republic

**Keywords:** freshwater reservoir, cold hypolimnetic layer, bacterivorous protists, new lineage of scuticociliates, protistan bacterivory rates

## Abstract

Deep, cold, and dark hypolimnia represent the largest volume of water in freshwater lakes with limited occurrences of phototrophs. However, the presence of prokaryotes supports populations of bacterivorous ciliates and heterotrophic nanoflagellates (HNF). Nevertheless, protistan bacterivory rates and the major hypolimnetic ciliate bacterivores are poorly documented. We conducted a high frequency sampling (three-times a week) in the oxic hypolimnion of a stratified mesoeutrophic reservoir during summer, characterized by stable physicochemical conditions and low water temperature. Using fluorescently labeled bacteria we estimated that ciliates and HNF contributed, on average, 30% and 70% to aggregated protistan bacterivory, respectively, and collectively removed about two thirds of daily hypolimnetic prokaryotic production. The ciliate community was analyzed by the quantitative protargol staining method. One scuticociliate morphotype dominated the hypolimnetic ciliate community, accounting for 82% of total ciliates and over 98% of total ciliate bacterivory, with average cell-specific uptake rate of 202 prokaryotes per hour. Moreover, long-amplicon sequencing revealed that the scuticociliate belongs to an unidentified clade closely related to the *Ctedoctematidae* and *Eurystomatellidae* families. The high-resolution sampling, microscopic, and sequencing methods allowed uncovering indigenous microbial food webs in the hypolimnetic environment and revealed a functional simplification of ciliate communities, dominated by a new bacterivorous scuticociliate lineage.

## Introduction

Bacterivory by heterotrophic nanoflagellates (HNF), ciliates, predatory bacteria [1, 2], and viral infection are believed to be the major factors balancing bacterioplankton production in the surface layers of both marine and freshwater environments [3–7]. In freshwaters, filter-feeding cladocerans, rotifers [8, 9], and mixotrophic flagellates [10–13] also contribute significantly to prokaryotic mortality in epilimnetic water layers. Protistan bacterivory in the epilimnion has been relatively extensively studied, in some cases even in a high temporal resolution to reveal short-lived maxima of fast-growing protists [14, 15]. In contrast, studies of protistan bacterivory in the hypolimnion remain rather rare and enigmatic, with a few exceptions of a limited number of measurements from a meromictic lake [16], or a study that focused mainly on flagellate bacterivory in a shallower mesotrophic lake [17].

Relatively little is known about the taxonomic, functional, and temporal dynamics of ciliates and their contributions to prokaryotic mortality rates in the deep and cold hypolimnetic layers. However, several studies indicated a decreased functional diversity of hypolimnetic ciliate communities [18–20], with enhanced proportions of primarily bacterivorous scuticociliate taxa [21–25]. For instance, in deep strata of Lake Traunsee (Austria) bacterivorous species accounted for up to 50% of the total ciliate abundance [23] and displayed significant relationships to that of hypolimnetic prokaryote abundances. Also, the ciliate assemblage structure along depths was significantly associated with abiotic depth gradients of temperature, conductivity, concentrations of dissolved organic carbon, total phosphorus, and oxygen [23].

In deep waters, the strong physiological effects of low temperatures influence the growth of hypolimnetic microorganisms dwelling in 4-6°C cold water, with an ~2.5-times decrease of microbial activities per a 10°C decrease in temperature (the Q10 coefficient [26]). Thus, hypolimnetic microorganisms should display ~4–6-times lower growth rates, compared to the typical doubling of prokaryotes and protists (in hours to days [27–31]) in epilimnetic water temperatures during summer stratification periods in temperate lakes. Additionally, deep hypolimnia represent completely dark environments limiting the presence of algivorous filter-feeding zooplankton, mixotrophic protists, and phototrophs. This implies that the role of rapidly growing epilimnetic phytoplankton-associated prokaryotes [28, 31, 32], profiting from labile organic substrates released by the autotrophs, is rather limited in the deep-water strata during the stratified period. Consequently, the lake hypolimnia, compared to the epilimnetic strata, host moderately abundant, but taxonomically and morphologically distinct bacterioplankton, frequently dominated by specific taxa of hypolimnetic bacteria and archaea [15, 33–38] that likely represent the prominent food resource for the hypolimnetic grazers. Such distinctive prey availability can also specifically shape the grazer communities, thus contributing to a functional simplification of hypolimnetic protistan communities towards the dominance of specialized bacterivorous or omnivorous protists [15, 19, 23, 39].

Recent studies indicate the coexistence of specific prokaryotic and protistan microbiomes, with distinct groups of nanoflagellates and prokaryotes in deep and cold hypolimnetic environments [15, 31, 36]. The latter studies are mainly based on short amplicon sequencing technologies which significantly improved our understanding of diversity and distribution of protists especially in combination with the CARD-FISH methodology [15, 39]. However, amplicon sequencing has proven to be more effective for HNF than for ciliates [15], for which short-amplicon approaches yield lower resolution than traditional morphological methods and fail to provide quantitative information, primarily due to the high number of rRNA gene copies [40–43]. Thus, whereas the high number of ASVs affiliated with ciliates have been recently reported in the reservoir hypolimnion, they frequently remained unclassified [15] due to the short length of 18S rRNA gene amplicon sequence. Shotgun metagenomics also have limitations for ciliate research [31] due to the difficulties of assembly of their large and complex genomes. Therefore, ecological studies on ciliates lack advances from sequencing and rely mostly on laborious morphology-based approaches.

We hypothesized that the stable environmental conditions, such as low water temperature, darkness, absence of phototrophs, and specific characteristics of hypolimnetic prokaryotes dominated by distinct taxa typical for oxic hypolimnia [15, 31, 33, 35–38] may select for cold-adapted and functionally specialized bacterivorous ciliate species. Because bacterivorous scuticociliates were found as prominent ciliate groups in hypolimnia of temperate lakes during midsummer periods [21, 23–25], we postulate that scuticociliates are the core hypolimnetic bacterivores significantly contributing to the bulk bacterioplankton loss rate. Therefore, to address the taxonomic diversity, trophic relationships, and temporal dynamics of ciliates in the understudied reservoir hypolimnion, we combined morphological analyses of ciliate communities by quantitative protargol staining [44], estimates of protistan bacterivory using fluorescently labeled bacteria [45], and long-amplicon sequencing [46]. Moreover, to cover possible short-lived peaks of rapidly doubling small bacterivorous protists [14, 15], we determined three-times a week abundances and the relative grazing impact of bacterivorous flagellates and ciliates. We focused particularly on ciliate species-specific bacterivory rates during the summer stratification phase in the hypolimnion of the meso-eutrophic freshwater Římov reservoir.

## Materials and methods

### Study site and sampling procedure

**Figure 1:**
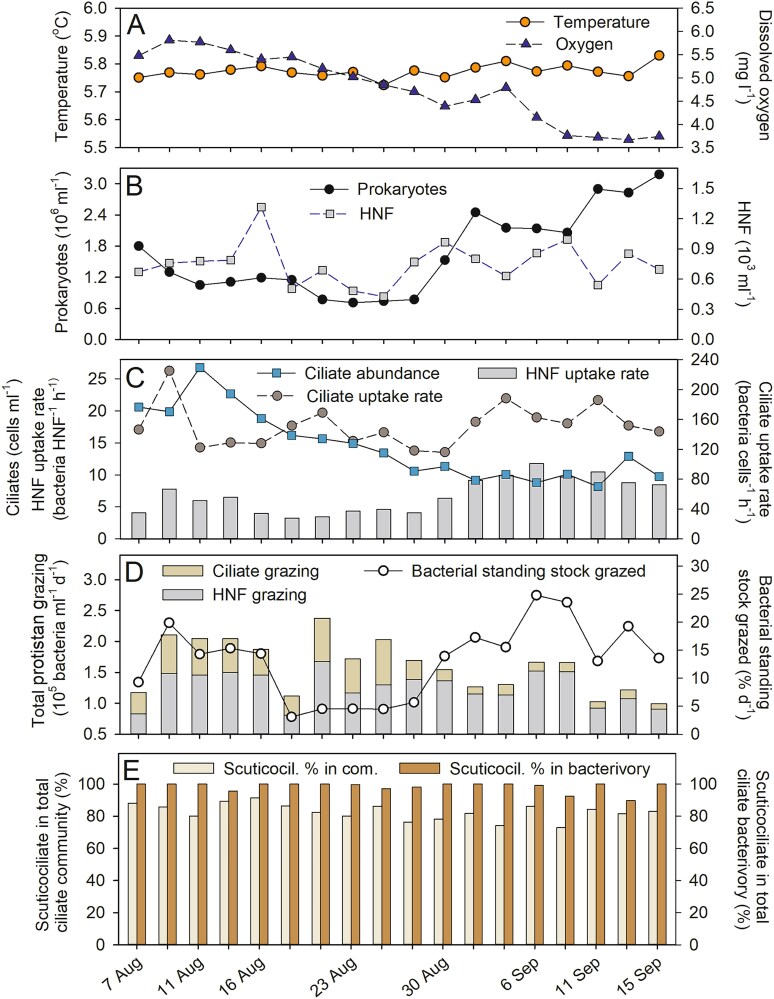
Basic physical, chemical, and microbial characteristics of the hypolimnion. Time-course changes (7 August–15 September) in the reservoir hypolimnion of: (A) temperate and oxygen concentrations; (B) abundances of prokaryotes and HNF; (C) abundance of ciliates and cell-specific uptake rates of ciliates and HNF; (D) total bacterivory rates of ciliates and HNF and a sum of proportions of prokaryote standing stocks removed daily by both protistan groups; and (E) proportions of the dominant scuticociliate (Scuticocil.) to total ciliates and to total ciliate bacterivory rates.

The reservoir Římov is a freshwater, mesoeutrophic, canyon-shaped, and circum-neutral water body situated in the South of the Czech Republic with surface area of 2.06 km^2^, volume 34.5 × 10^6^ m^3^, and an average retention time of 85 days [47]. Approximately 60% of the reservoir watershed is forested, the rest of the area consists of an extensive agricultural land with smaller settlements situated in a hilly countryside. The whole watershed is designated as a state-protected area for the accumulation of drinking water with strict measures of nutrient loading control of the reservoir inflow water quality. Recreation activities are forbidden in the reservoir and within a “protective country belt” surrounding the reservoir banks as previously described [47, 48]. The river inflow is 13.5 km distant from our sampling station, which is situated above the deepest point at the lacustrine reservoir part (max. depth 43 m). To achieve a high temporal resolution characterizing typical microbial community dynamics and doubling times, we conducted high-frequency sampling (three-times per week) from the oxic hypolimnion (25 m depth) during the period of 7th August to 15th September 2023. This frequency was chosen as a balance between logistical feasibility and capturing expected short-term peaks in the studied microbial populations. In total, 18 samples were collected, representing the most stable period during water column stratification [47]. The depth of 25 m represented dark hypolimnetic water strata with stable physico-chemical properties, high concentrations of inorganic nutrients (Figs. S1, and S2), stable temperatures (5.7 – 5.8°C), and a slow decreasing trend in oxygen saturation (Fig. 1A). Samples were taken with a Friedinger sampler, prefiltered through a 200-μm-mesh-sized plankton net, collected in pre-washed plastic barrels and split into subsamples for processing with different methods (see below).

### Basic chemical background data and chlorophyll-*a* determination

Basic physical and chemical parameters – water temperature, pH, dissolved oxygen, and oxygen saturation was measured with a multiparametric probe YSI EXO2 (Yellow Springs Instruments, OH, USA). Chlorophyll-*a* (Chl-a) concentrations were measured with a submersible fluorescence probe (FluoroProbe, bbe-Moldaenke, Kiel, Germany) at 1 m intervals in the upper 30 m of a water column. Total dissolved organic carbon was determined with the FormacsHT Analyzer (Skalar, Analytical B.V., The Netherlands). Total phosphorus, dissolved reactive phosphorus, and dissolved nitrogen were determined via flow injection analysis with spectrophotometric assignment using a FIAstar 5012 (Foss, Denmark).

### Prokaryotic and protistan abundances and cell sizing

Formaldehyde-fixed samples (2% final concentration) were used to enumerate prokaryotes and HNF on 0.2-μm and 1-μm pore-size filters (Osmonics, Livermore, CA), respectively [45]. Ciliates were counted in samples fixed with the Lugol-formaldehyde-thiosulphate method [49]. All samples were stained with DAPI (4′-6-diamidino-2-phenylindole), and microorganisms were counted via epifluorescence microscopy (Olympus BX53; Tokyo, Japan) under 1000× (prokaryotes and HNF) and 600× magnifications (ciliates). To estimate the prokaryotes’ mean cell volume (MCV), cells (>300) were sized by using a semiautomatic image analysis system (NIS-Elements 5.1., Laboratory Imaging, Prague, Czech Republic) as previously described [14]. To calculate MCV of hypolimnetic scuticociliates (approximated to prolate spheroids), lengths and widths of 100 cells were measured manually on-screen with a built-in tool of an image analysis system (NIS-Elements 5.1, [29]). The average sizes of the hypolimnetic ciliate and prokaryotic cells were used to estimate a doubling time of the bacterivorous scuticociliate species, assuming prokaryotes to be the sole carbon source for its growth. We used the ratio between the total prokaryote prey biovolume consumed per day and the mean ciliate cell biovolume (as % of cell volume, corresponding to the prokaryote biovolume consumed per day, representing thus volumetric gross growth efficiency), assuming 30% to 40% gross growth efficiencies as a typical range of values reported for bacterivorous protists [29, 50].

### Estimation of hypolimnetic bacterioplankton growth rates

At two occasions, on 11th and 25th August 2023, the maximum growth rates of hypolimnetic bacterioplankton were estimated in biological triplicate experiments. Hypolimnetic water was filtered through 1-μm membrane filters and incubated in the dark at 5.8°C temperature.

Prokaryotic abundance was determined daily for a period of 6 days. The bacterioplankton growth rates were calculated using ln-transformed data of bacterioplankton abundance with linear regression as the slope of the best-fit line over three days with the exponential growth.

### Tracer technique to estimate flagellate and ciliate bacterivory

Flagellate and ciliate bacterivory rates were estimated using fluorescently labeled bacteria (FLB) following the published protocol [51]. The FLB were prepared from a mixture of strains of highly abundant bacterioplankton taxa in lakes from the genus *Limnohabitans* and *Polynucleobacter* [52] isolated from the reservoir: *Limnohabitans planktonicus* (short rods, MCV of 0.135 μm^3^, [53]) and one undescribed strain (czRimov8-C6) from the PnecC lineage of *Polynucleobacter* (short curved rods, MCV of 0.054 μm^3^, [29]). The strains were mixed in a numerical ratio of 1:1, fluorescently labeled [51], and frozen. Our FLB stock solution contained cells well mimicking typical sizes of bacterioplankton in the reservoir hypolimnion (MCV 0.08 μm^3^). Prior to be used, FLB were thawed and resuspended via sonication pulses. The uptake of suspended tracer FLB thus indicates mainly the ability of protists to feed efficiently on suspended prokaryotes, whereas it may not reflect precisely grazing of protists on the particle-attached prokaryotes. Grazing experiments were conducted at *in situ* temperature and the proportions of the tracer FLB amount added and calculations of cell-specific uptake rates were conducted as previously described in detail [54]. Prey ingestion rates are considerably slower in low water temperatures (Fig. 1A), thus we prolonged the incubation times (10-min for ciliates and 60-min for flagellates) and enhanced the tracer amount added, yielding 16–20% of total prokaryotes [54]. These incubations under *in situ* temperature yielded well countable tracer amounts of 1–5 FLB per HNF cell and 1–15 FLB per ciliate. The incubations were terminated with the Lugol-formaldehyde-thiosulphate fixation for both flagellates and ciliates, preventing egestion of ingested prey from food vacuoles [49]. All samples with FLB tracers were processed within 2-8 hours after the incubation with FLB to limit any bias of our data related to sample storage. Subsamples were stained with DAPI, concentrated on 1-μm black filters (Osmonics), and the FLB number in food vacuoles of HNF and ciliates were quantified via epifluorescence microscopy [14, 45]. To estimate the total protistan grazing, we multiplied average prokaryotic uptake rates (based on tracer FLB uptake) of ciliates and HNF by their *in situ* abundances.

### Evaluation of ciliate community composition

**Figure 2:**
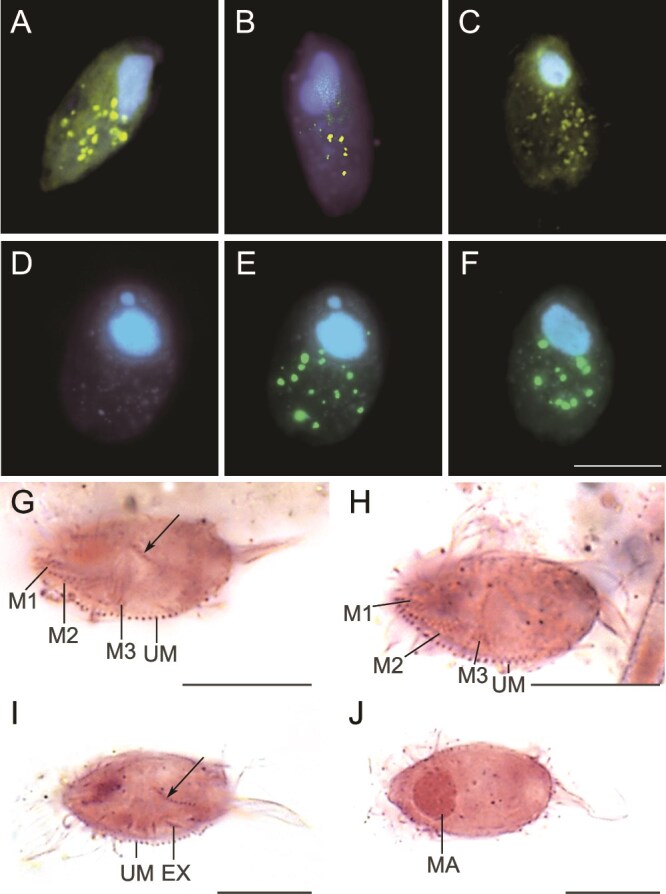
Microphotographs of the undescribed scuticociliate. Epifluorescence microscopy images after DAPI and DTAF staining (A–F) and from bright field microscopy after protargol impregnation (G–J). Overlay Z-stack images of DAPI-stained ciliate cells show the characteristic ellipsoidal shape and size of the cells, the macronucleus (in blue) and ingested FLBs in food vacuoles (in yellow, A–C, E, F). Images D and E exemplify a pair of corresponding images of a DAPI-stained cell without visible FLBs (D) and with ingested FLBs (E). Morphological details such as the prominent undulating/paroral membrane with the characteristic falciform end (arrow), extrusomes, the macronucleus, and (probably several) caudal cilia are shown (G, H). Details of the arrangement of the oral ciliature including M1-M3 and the undulating/paroral membrane are presented in I and J. CC – Caudal cilium/cilia, EX – Extrusomes, MA – Macronucleus, M1 – Adoral membranelle 1, M2 – Adoral membranelle 2, M3 – Adoral membranelle 3, UM – Undulating/paroral membrane. Arrows point to the characteristic hook-like/falciform posterior end of the undulating/paroral membrane. Scale bars 10 μm.

The ciliate community structure was evaluated from both DAPI-stained samples under epifluorescence microscopy [45] and quantitative protargol stained samples (QPS) fixed with Lugol’s solution and postfixed with Bouin’s fluid. Briefly: for QPS preparations, we filtered 30 ml of fixed samples onto 0.8-μm pore-size nitrocellulose filters (with counting grids, Sartorius, Auburn, USA) and QPS was performed following the previously described protocol [55], with modifications described elsewhere [44]. Using light microscopy, we inspected the whole filter area at magnifications of 600× or 1000× (Olympus BX50, Optical, Japan). Each ciliate cell was taxonomically identified to species, genus or morphotype level (99% of the total ciliates) by using the taxonomic keys [56–58]. We inspected 100–200 individuals per sample. For more details on the above approaches and criteria used for ciliate identification, see references [56–59]. Because protargol staining (Figs. 2G–J and S4) was applied in parallel with epifluorescence microscopy for ciliates, the major bacterivorous and non-bacterivorous ciliate taxa in the hypolimnetic samples were identified to species or at least morphotype levels (Table 1). In epifluorescence microscopy we also used additional criteria, such as ciliate cell size, the position and size of nuclei, and the way the FLB tracers were arranged in food vacuoles [45]. The grazing data of the same taxa from the whole study period were pooled to calculate cell-specific grazing rates at the species or morphotype levels. Moreover, from the dominant scuticociliate, cell morphometric data were collected from the QPS-preparations to characterize and taxonomically assign this particular morphotype.

**Table 1:** Hypolimnetic bacterioplankton growth rate and doubling time (mean values ± SD of triplicate treatments) and the proportion of the prokaryotic production removed by total protists (ciliates and HNF) per day. All parameters were measured under *in situ* temperature (5.8°C).

Date	Prokaryotic growth rate	Prokaryotic doubling time	Prokaryotic production
	(day^−1^)	(hours)	removed by protists
	Mean ± SD	Mean ± SD	(% day^−1^)
11 August, 2023	0.136 ± 0.008	122 ± 7.5	62.5
25 August, 2023	0.116 ± 0.002	144 ± 2.0	73.8

### Biomass collection, DNA extraction, and sequencing analysis of long amplicons

Samples (up to 2 l) were prefiltered through 40 μm mesh to remove large phyto- and zooplankton and then the biomass was collected on 0.22 μm pore-size polyethersulfone membranes (MILLIPORE EXPRESS, Darmstadt, Germany). The filters were stored in cryovials with 1 ml of DNA/RNA Shield at −80°C*.* Prior to DNA isolation, thawed filters were cut into 3 mm squares and digested in their storage solution with lysozyme (final concentration 20 mg ml^−1^) for 1 h at 37°C, followed by treatment with 20 μl proteinase K (20 mg ml^−1^), and 20 μl SDS 20% for 1 h at 56°C. DNA isolation was carried out with the Quick-DNA High Molecular Weight MagBead Kit (Zymo Research, California, USA) according to the manufacturer’s instructions. DNA was quantified using the Qubit 3.0 Fluorometer (Invitrogen, Carlsbad, California, USA) with the dsDNA HS Assay Kit (ThermoFisher Scientific, Massachusetts, USA). Extracted DNA was sent to the Rush University Genomics and Microbiome Core Facility (Chicago, Illinois, USA) for sequencing. There, the forward primer V4F (5’-CCAGCASCYGCGGTAATTCC-3′, [60]) and reverse primer 21R (5’-GACGAGGCATTTGGCTACCTT-3′, [61]) were used to amplify 18S, ITS, and part of the 28S rRNA gene regions. These primers were identified as the most robust combination for generating long amplicon libraries for protistan communities [46], although they exhibit slight biases against the *Hacrobia* supergroup.

An equimolar library was prepared using Fluidigm barcodes, followed by PacBio library preparation with the SMRTbell system for high-fidelity long-read sequencing. Sequencing was performed on a Sequel II platform using a SMRT 8 M cell with a 30 h movie runtime. The resulting sequences were demultiplexed and dereplicated at the Rush University Research Bioinformatics Core Facility using DADA2 script specifically designed for PacBio long-amplicon data [62]. Chimeric sequences were identified and filtered using the BimeraDenovo algorithm, with a minimum fold parent-over-abundance threshold set to 3.5. Sequences representing nonchimeric ASVs were taxonomically assigned using 18S rRNA gene fragment identified with Barrnap software (https://github.com/tseemann/barrnap). The PR2 database 5.0.0 [63] was used as the reference for taxonomic assignment.

### Phylogenetic analysis

All sequences affiliated with the *Pleuronematida*, a group of the *Scuticociliatia* were selected for the phylogenetic analysis. Additionally, all entries affiliated with *Scuticociliatia* covering the corresponding region of 18S rRNA gene were extracted from the 18S rRNA databases PR2 release 5.0.0 and SILVA release 138.1 [64]. Duplicate sequences with identical accession numbers were removed. The alignment of sequences (n = 251, 190 unique) was performed using MAFFT version 7.526 [65–67] with default settings on the server. The resulting alignment was manually inspected, and 1281 nucleotide positions were retained for the tree construction in IQ-TREE version 2.1.2 [68]. Maximum likelihood (ML) analysis was conducted with 1000 bootstrap replicates using the ultrafast bootstrap algorithm. The best-fit model TIM2 + F + R4 was selected by ModelFinder according to Bayesian Information Criterion. Bootstrap values and approximate Bayes parametric test results were used to assess the branch support. The tree was visualized using the ITOL online tool [69], and its topology was compared with the recent taxonomic studies on scuticociliates [70–72], with relevant taxonomic names adopted accordingly.

## Results

### Basic physical, chemical, and microbial characteristics of the hypolimnion

The water column of the Římov reservoir was thermally stratified throughout the study period, resulting in distinct vertical profiles of temperature, oxygen, Chl-a, and penetration of photosynthetically active radiation (Fig. S1). Compared to the nutrient-limited epilimnion (0.5 m depth), the hypolimnetic depth showed high concentrations of inorganic nutrients (Fig. S2). Temperatures in the surface layer ranged from 18.5°C to 24.5°C (Fig. S3) compared to low and stable water temperatures (5.75–5.83°C, Fig. 1A) in the hypolimnion at 25 m depth. Although the epilimnetic waters were largely oxygen saturated (8.2–11.2 mg l^−1^), the hypolimnetic oxygen concentrations were considerably lower and gradually decreased from 5.8 to 3.8 mg l^−1^ towards the study end (Fig. 1A). Chlorophyll-*a* concentrations in the hypolimnion were negligible between 1.0–1.5 μg l^−1^, corresponding rather to a natural background of the method sensitivity, than to any substantial phytoplankton occurrence. Microscopic sample inspections indicated they were most likely related to the frequent occurrence of decaying remains of colonial diatoms (*Fragilaria* sp.), much less of dead *Staurastrum* cells, and extremely rare small flocks of *Microcystis*. However, almost no vital algae with Chl-a autofluorescence were observed. In contrast, the average epilimnetic (1 m depth) Chl-a concentration was 10.1 μg l^−1^, ranging from 5.3 to 18.8 μg l^−1^ during the study period (Fig. S3).

### Microbial abundance and protistan bacterivory rates

In the hypolimnion, prokaryotic densities dropped from initial 1.8 × 10^6^ ml^−1^ to 0.71 × 10^6^ ml^−1^ in late August and then sharply increased in mid-September, peaking at 3.18 × 10^6^ cells ml^−1^. HNF showed one conspicuous abundance peak (1.31 × 10^3^ cells ml^−1^, Fig. 1B) on 16^th^ August, dropped to a minimum of 0.45 × 10^3^ cells ml^−1^ in late August, and then oscillated within a relatively narrow range of 0.54–0.99 cells × 10^3^ ml^−1^ till the end.

Ciliates reached their maximum abundance of 26 cells ml^−1^ (Fig. 1C) on 11^th^ August, then continuously decreased till 21^st^ August and stayed low within 8 to 12 cells ml^−1^ until the study end. Uptake rates of FLB (see examples in Fig. 2) by individual protistan groups allowed estimating cell-specific uptake rates on the community level of all ciliates and HNF (Fig. 1C). Ciliates and HNF contributed, on average, 30% and 70% of aggregated protistan bacterivory rates, with individual cell-specific uptake rates ranging from 108 to 222 prokaryotes ciliate^−1^ h^−1^, and 3.5 to 11.8 prokaryotes HNF^−1^ h^−1^, respectively. Taking into account the prokaryote abundance, the aggregated protistan grazing would daily remove, on average, 12.3% of the hypolimnetic prokaryotic standing stocks (Fig. 1D). To estimate the overall impact of protistan bacterivory on bacterioplankton dynamics, we estimated net growth rates of the hypolimnetic bacterioplankton under *in situ* temperature on August 11^th^ and 25^th^. It ranged from 0.116 to 0.136 day^−1^, yielding doubling times of 122 and 144 hours (Table 1). When these production data are confronted with the daily aggregated protistan bacterivory rates of both HNF and ciliates, the protists eliminated 62.5% and 73.8% of the daily hypolimnetic prokaryotic production in these two occasions, respectively (Table 1).

### Scuticociliates—prominent hypolimnetic ciliate bacterivores

**Figure 3:**
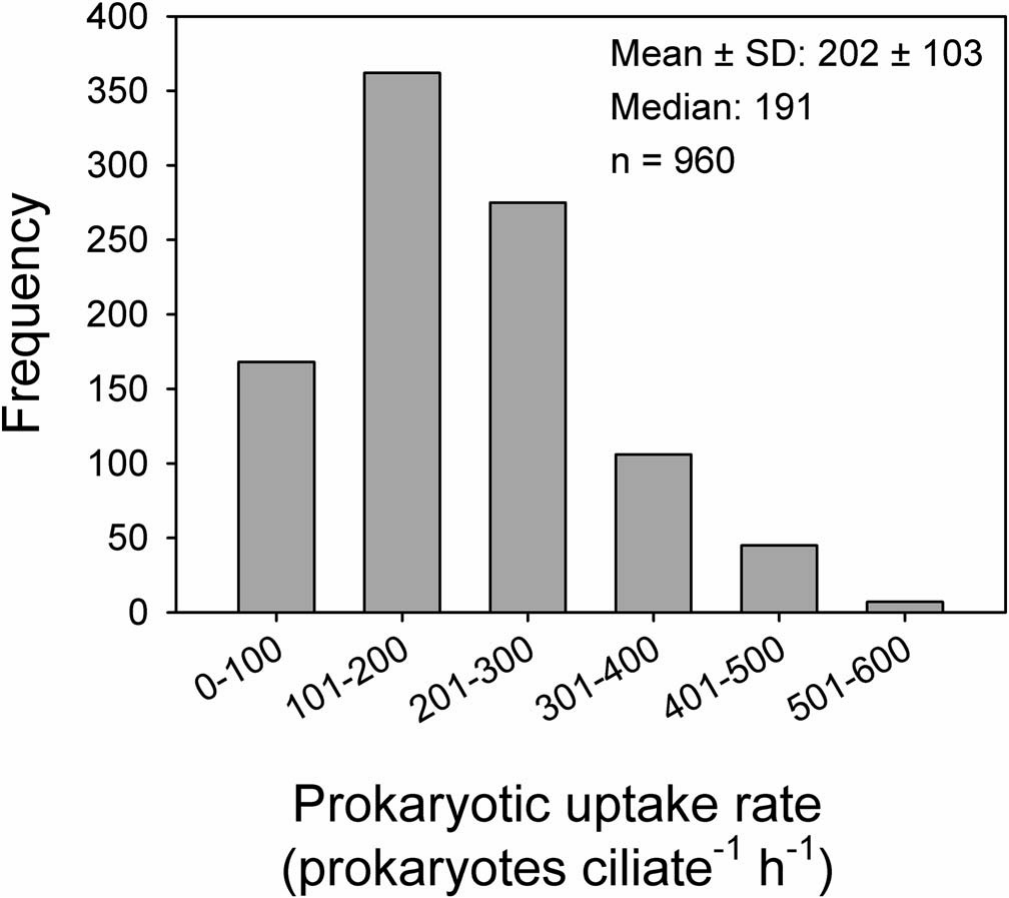
Uptake rate of prokaryotes by the dominant scuticociliate. A size class distribution of cell-specific uptake rates of the dominant scuticociliate with inserted overall mean and median values based on the tracer FLB approach and inspecting 960 individual cells of the scuticociliate.

The relatively narrow range of the ciliate cell-specific bacterivory rates (Fig. 1C) suggested that the ciliate bacterivore community was dominated by one scuticociliate morphotype (Fig. 2A-J), forming, on average, 82% of total ciliates and over 98% of total ciliate bacterivory. Based on the 960 inspected individuals of the scuticociliate, the mean (± SD) uptake rate of 202 ± 103 prokaryotes ciliate^−1^ h^−1^ was very close to the median value (191 prokaryotes ciliate^−1^ h^−1^), with a relatively narrow scatter of prokaryotes uptake rates (Fig. 3). More than 70% of the uptake rates fall within two uptake rate classes, i.e. from 101–200 and 201–300 prokaryotes ciliate^−1^ h^−1^, whereas only around 1% of the population had uptake rates higher than 500 prokaryotes ciliate^−1^ h^−1^. We sized the hypolimnetic prokaryotes in 12 samples over the summer period, yielding prokaryotic MCV (± SD) of 0.081 ± 0.02 μm^3^. Considering the mean uptake rates of the scuticociliate (Fig. 3), the total biovolume of prokaryotic prey ingested per day compared to the ciliate MCV (661 μm^3^), and assuming a gross growth efficiency of bacterivorous protists within the range of 30%–40%, the ciliate bacterivory rate might support *in situ* doubling times of the scuticociliate at a range of 98–132 h.

### Community composition of ciliates in hypolimnion

Using FLB tracer and the QPS techniques, the ciliate assemblages were analyzed to the genus or species level (Fig. S4) along with their capabilities to take up prokaryotic particles (Fig. 2 and Table 2). The ciliate community was clearly dominated by one scuticociliate morphotype (82% of total ciliates). We also detected three other ciliate taxa with uptake of FLB, generally bacterivorous or omnivorous species such as *Vorticella* sp., *R. brachykinetum*, and *Halteria* sp. (Fig. S4). Besides, we detected nine ciliate taxa without any FLB uptake, but only predatory *Mesodinium* spp. and the algivorous prostome *Urotricha globosa* contributed significantly to the total community composition (6.0% and 4.8%, respectively). Other non-bacterivorous species detected (Table 2), represented mostly detritivores or eventually predatory species (Fig. S4). However, proportions of none of these species exceeded 0.3% of the total ciliates. Overall, less than 1% of the total ciliates could not be identified to the genus or morphotype level (Table 2).

**Table 2:** Ciliate species detected in the hypolimnion of the Římov reservoir, cell-specific bacterial uptake rates (mean ± SD) in bacterivorous and omnivorous species and relative proportion of species on the total ciliate community. Note: The undescribed scuticociliate – this new lineage is presented in phylogenetic tree as the “Group detected in this study” (for details see the phylogenetic tree in Fig. 4).

Species with bacterial uptake				
**Class**	**Species**	**Bacterial uptake rate**	**Ciliate feeding mode**	**Proportion in community (%)**
		**bacteria cell** ^**−1**^ **h**^**−1**^		
		**(mean ± SD)**		
**Oligohymenophorea**	Scuticociliate undescribed	202 ± 103	bacterivorous	81.8
	*Vorticella* sp.	775 ± 88	bacterivorous	0.3
**Spirotrichea**	*Halteria* sp.	324 ± 71	omnivorous/bacterivorous	3.4
	*Rimostrombidium brachykinetum*	43 ± 36	omnivorous	2.4
**Species without bacterial uptake**				
**Litostomatea**	*Mesodinium* sp.	–	predatory	6.0
	*Actinobolina smalli*	–	predatory	0.02
**Prostomatea**	*Urotricha globosa*	–	algivorous	4.8
	*Balanion planctonicum*	–	algivorous	0.1
	*Coleps hirtus*	–	detritivorous	0.2
**Oligohymenophorea**	*Stokesia vernalis*	–	algivorous	0.3
	*Cinetochilum margritaceum*	–	detritivorous	0.2
	*Astylozoon fallax*	–	detritivorous	0.1
	*Cyrtolophosis mucicola*	–	detritivorous	0.1
**Colpodea**				

	Not identified	–	Not determined	0.3

**Figure 4:**
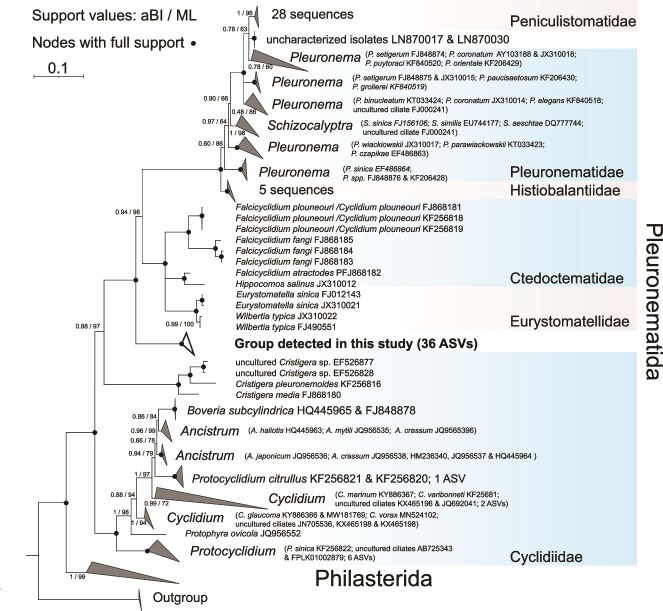
Phylogenetic tree of scuticociliates. Maximum likelihood tree constructed from 261 partial SSU rDNA sequences (1281 nucleotide positions), highlighting the newly detected group of ciliates (in bold). Numbers on branches represent approximate Bayes test values (aBI) and bootstrap support from maximum likelihood (ML). Fully supported nodes are indicated by black circles. The outgroup is represented by members of genus *Colpoda* (*Ciliophora*, *Colpodea*). The tree scale corresponds to 0.1.

### Sequence and phylogenetic analysis of scuticociliates

We identified 45 amplicon sequence variants (ASVs) from long-read amplicons (~4200 bp) affiliated with the order *Pleuronematida* within the *Scuticociliatia*, based on the 18S rRNA gene region (Fig. 4). Phylogenetic analysis revealed that 36 of these ASVs formed a distinct clade, robustly separated from previously reported sequences, with high bootstrap and approximate Bayesian support values. This distinct separation prevents us from affiliating the identified cluster with any described families (Fig. 4). However, the complex and non- monophyletic organization within *Pleuronematida*, primarily based on morphological descriptions, makes it challenging to determine whether the group detected in this study represents a new family.

### Morphological identification of the dominant scuticociliate

The morphological inspections of the scuticociliate from the QPS preparations and under epifluorescence microscopy did not allow to unambiguously identify the species (Fig. 2A-J). However, some morphometric measurements and counts were possible and are as follows (n = 21 if not stated otherwise): cells narrowly ovate with truncated apical plate; size (± SD) 17.1 ± 1.0 × 9.0 ± 1.2 μm on average in protargol preparations and 17.1 ± 1.4 × 8.5 ± 1.3 μm in epifluorescence microscopy (n = 100) with a biovolume of 661 ± 232 μm^3^ (calculated from the latter measurements); length:width ratio 1.9:1; one roughly ellipsoidal macronucleus of 4.4 ± 0.7 × 3.1 ± 0.6 μm; one ellipsoidal micronucleus of 1.7 ± 0.7 × 1.1 ± 0.4 μm, adjacent or close to the macronucleus; nuclei in posterior third of the cell, micronucleus located anterior of macronucleus; extrusomes rod-like to slightly fusiform, sparse, size about 1.2 ± 0.2 μm on average (n = 9); contractile vacuole terminal; approximately nine or 10 longitudinal somatic kineties; one long caudal cilium of 11.2 ± 1.1 μm in length (n = 11), probably accompanied by more and shorter caudal cilia of 7.9 ± 1.3 μm in length (n = 10). However, the latter were not clearly distinguishable from the somatic cilia and the usually long cilia from the undulating/paroral membrane; buccal field prominent with a paroral membrane of 14.6 ± 1.0 μm in length, cell length:length of paroral membrane ratio of 1.2:1, occupying 89% of cell length on average (n = 10), paroral membrane distinctly curved at posterior end and hook-like as is characteristic for the scuticociliate genus *Falcicyclidium*; adoral membranelles 1–3 not clearly visible but different from yet described *Falcicyclidium* species (Fig. 2G-J).

## Discussion

### Hypolimnion hosts functionally simplified ciliate communities

Microscopy facilitated estimates of taxon-specific bacterivory rates (Table 2) and documented that stable hypolimnetic layers were dominated by a functionally simplified ciliate community with the dominance of one undescribed bacterivorous scuticociliate (Figs. 1 and 4). Also, theoretical models and empirical data propose a steep decline in species richness and respiration rate with low temperatures among different ciliate species leading to a simplification of the microbial food web [73]. These findings are in line with considerable impacts of extreme or specific environmental conditions on the plankton community composition in aquatic habitats, as has been reported for cold Arctic lakes [74], high mountain lakes [75, 76], high salinity lakes [77], or extremely acidified lakes [78]. Such circumstances significantly reduce taxonomic and functional diversity and simplify microbial food webs to a few well-adapted protistan species [73, 74, 78]. This corroborates our finding that currently even the undescribed lineage of the bacterivorous scuticociliate, with the relatively narrow scatter in cells-specific uptake rates (Fig. 3), entirely dominated the ciliate community and also its bulk bacterivory rates (Fig. 1E). In contrast, examples of cells-specific bacterivory rates measured for *Halteria* sp. in the warmer reservoir epilimnion [54, 79], or of other ciliates taxa in hypertrophic shallow lakes [45] indicate that the bacterivory rates of a particular ciliate species growing more rapidly in the epilimnetic conditions vary more broadly (likely reflecting more rapid oscillations in their physiology or cell cycle) than in the cold hypolimnion.

Compared to the typical epilimnetic ciliate communities [23, 57, 59, 80], ciliate species with predatory, detritivorous or algivorous feeding modes were far less represented in the reservoir hypolimnion (Table 2, Fig. S4). Moreover, even broadly accepted assignment of the feeding mode of highly abundant prostomes as algivorous is terminologically rather misleading. For instance, the genus *Urotricha* (ca. 5% of the hypolimnetic ciliates) is commonly considered to be a typical algivore in the epilimnetic layers [23, 57, 59, 81–83], but in fact it is primarily a flagellate hunter that feeds efficiently on both plastid-bearing as well as on heterotrophic flagellates [30, 82]. Bacterivorous HNF are the most abundant hypolimnetic protist group [15] (Fig. 1B), thus representing an important food source for flagellate grazers such as *Urotricha* spp., due to the absence of mixotrophic flagellates and other algae (see Chl-*a*, Fig. S1).

The trend observed for hypolimnetic ciliate community dynamics contrasts to sunlit lake water layers with the prominent role of primary producers. This is also reflected in a more diverse epilimnetic ciliate community composition and considerably enhanced proportions of algivorous, raptorial, and omnivorous species of the genera *Balanion*, *Strobilidium*, *Rimostrombidium*, *Halteria*, *Askenasia*, *Urotricha*, and *Mesodinium* [23, 57, 59, 80, 84].

However, the role of major protist groups and their prevailing feeding modes in the cold hypolimnetic water layers are less documented compared to the more productive epilimnetic waters. Recent studies on the vertical distribution of flagellated protists in the reservoir (based on microscopic and sequencing approaches) clearly indicated a distinct community composition of hypolimnetic HNF assemblages, being dominated by parasitic and heterotrophic groups such as *Perkinsozoa*, *Cercozoa*, *Kinetoplastida*, and *Telonemia* [15, 85]. However, limited morphological characteristics and availability of suitable FISH probes targeting the major HNF groups hinder the progress in understanding their diverse feeding modes and trophic roles in microbial food webs [15, 39, 86].

In contrast to HNF, the morphologically diverse ciliates offer an alternative and sufficiently robust base of distinctive morphological features for reliable species or morphotype identification [56–58], which can be supported by molecular approaches. In our study, this allowed significant refinements of the taxonomic resolution at the level of scuticociliates and revealed their prominent new lineage (Figs. 2 and 4). The combination of QPS with the tracer FLB technique unveiled that this scuticociliate was responsible for approximately one third of prokaryotic mortality in the hypolimnion (Fig. 1D and E). Such a high contribution of ciliate bacterivory has rarely been observed in the epilimnetic layers, which were mostly dominated by flagellate bacterivory [12, 17, 87, 88]. However, hypertrophic shallow lakes, hosting extreme abundances of bacterivorous ciliates, represent exceptions from this general pattern [45, 89].

### Scuticociliate identification is challenging

For an unambiguous identification of small scuticociliates to genus or species level, both the arrangement of few single basal bodies of the tiny structure called “scuticus” and the adoral membranelles need to be recognized in detail. Without specific silver stains revealing these structures, it is impossible to determine a morphospecies in most cases [90]. Moreover, *Cyclidium*-like ciliates from different genera have a very similar overall morphology and ciliary patterns and are hard to discriminate from one another especially in field samples. Without having a culture of the desired species—as in our case here—to observe the living cells and additionally stain specific characteristics, the unambiguous identification of a scuticociliate remains doubtful. Despite using QPS preparations (Figs. 2G–2J and S4) and epifluorescence microscopy on field samples, we were unable to identify the most abundant hypolimnetic scuticociliate. However, this ciliate possessed one feature typical for *Falcicyclidium*, which is the prominent hook-like (falciform) paroral membrane that is conspicuously curved at the posterior end and not found in such peculiar shape in any other *Cyclidium*-like genus [91, 92]. However, we could not clearly see the multiple caudal cilia, the exact patterns of both the three adoral membranelles and the scuticus. Moreover, *Falcicyclidium* species have yet only been recorded from marine habitats [91, 92]. Taken together, our data are consistent with assigning the scuticociliate to a new lineage (Fig. 4), closely related to *Falcicyclidium*, *Wilbertia*, and *Eurystomatella* [93], but a more detailed description, including an integrative taxonomic approach from morphology and molecular sequencing from clonal cultures, is needed to verify the identity [82]. The rather distant position of the lineage representing 36 ASVs in the phylogenetic tree (Fig. 4) indicates that the group found in our study might represent even a new family of scuticociliates. The detection of this group enabled the identification of tightly related and previously unassigned ASVs in the reservoir hypolimnion following the onset of the water-column stratification in 2016 [15], highlighting broader relevance of this lineage. Moreover, small bacterivorous scuticociliates are frequently found even as prominent groups well adapted to extreme environments of hypolimnetic anoxic zones of lakes [24, 25, 94]. Their taxonomy still requires refinements, making the description of new species from hypolimnetic environments quite likely.

### Tracing ciliate species and their abundance from sequencing datasets

DNA sequencing-based approaches displaying protist diversity, biogeography or distribution along seasons or depths in a waterbody has become a widespread approach. However, estimating the abundance of individual taxa from such datasets based on OTU or ASV read counts do not correspond to the actual number of a ciliate morphospecies in a water sample because of the considerably varying and often unknown number of SSU rRNA gene copies present in a species [41, 42, 95]. Consequently, amplicon sequencing does not reflect the actual morphotype abundance in a water body although some general patterns or presence/absence may be recognized [82, 96, 97]. Moreover, a prerequisite for analyzing distribution patterns along time and depth gradients is that a species-specific sequence of the respective ciliate is available in public databases, which is not the case for most of them due to prevalence of studies based only on morphology. Therefore, integrating morphological analyses with sequencing approaches is essential to enhance our understanding of ciliate diversity and ecology.

### Broader ecological implications of microbial food web dynamics in hypolimnia

Our results indicate slow to moderate doubling times of protistan and prokaryotic populations in cold hypolimnetic layers. On average, aggregated HNF and ciliate bacterivory removed 12.3% of hypolimnetic prokaryotic standing stocks daily (Fig. 1D). Thus, the grazing-induced mortality would remove the bacterioplankton standing stock in 195 hours (=8.1 days). Our two estimates of hypolimnetic bacterioplankton growth rates yielded doubling times of 122 and 144 hours, respectively, which implies that protistan grazing would control ca. 63% and 74% of the daily prokaryotic production (Table 2). Moreover, the latter values also implicate that 26%–37% of the prokaryotic production were controlled by other sources unrelated to protistan grazing. Because zooplankton is practically absent in the hypolimnion [98], the most likely source of the remaining bacterioplankton losses were virus-induced cell lysis, known to cause between 20–50% of total prokaryote mortality in deeper strata of freshwater lakes [7], or perhaps also predatory bacteria [1, 2].

Growth potential of the hypolimnetic prokaryotes (Table 1) and their considerable mortality rates induced by protists suggest that the large volume of oxic hypolimnetic waters represents the space for intense prokaryote-protistan trophic interactions and a significant accumulation of nutrients during the stratification period (Fig. S2). Although our measurements refer to one particular hypolimnetic depth (25 m), yet it provides important insights into the rarely quantified rates of carbon fluxes in microbial food webs under specific hypolimnetic conditions. Small bacterivorous HNF and ciliates controlled a considerable amount of the hypolimnetic bacterioplankton production (Fig. 1 and Table 1), whereas, almost no grazers of these protists were observed, except for some predatory ciliates such as *Mesodinium* and *Urotricha* (Table 2). Co-occurrence network analyses of epi- and hypolimnion of two lakes identified different prey items of *Urotricha castalia*, *Halteria grandinella*, and *Coleps viridis*, implying the potential flexibility of ciliates to exploit various food resources related to the environment [96, 99, 100]. Due to the lack of significant amounts of other prey items than prokaryotes and perhaps small HNF, the functionally simplified hypolimnetic food webs were dominated by protistan bacterivores (both ciliates and flagellates [15, 101, 102],). However, these trends were particularly apparent in the dominance of bacterivorous scuticociliates in hypolimnia of deep lakes [19, 20, 23].

Our results have brought more detailed insights into turnover rates of hypolimnetic microbial communities (doubling times ca. 4-6 days) that along with a limited matter transfer to higher trophic levels result in a considerable accumulation of inorganic nutrients in the deep water strata, prior being mixed with the rest of the water column during fall. This scenario supports more general views of the deep oxic water layers as cold, dark, and nutrient-rich (Fig. S2) environments, selecting not only for the specific ciliate bacterivores (Fig. 2), but also for typical functional groups of prokaryotes found in deep lakes and the reservoir such as nitrifiers (*Nitrosospira* spp., ammonia-oxidizing archaea), methylotrophs (LD28, *Methylobacter*), clades of *Planctomycetota*, and *Verrucomicrobiae*, or cold-adapted freshwater lineages of *Methylopumilus* (*Methylophilaceae*), *Rhodoferax*, and *Limnohabitans* genera [15, 35, 36, 103].

The scuticociliate lineage detected (Fig. 4) is likely more broadly distributed in the hypolimnion. For instance, after the onset of the reservoir stratification in late May 2016, a ciliate sequence (ASV 2032) was already detected in the hypolimnion. It was initially identified as unclassified *Ciliophora* [15], but now we detected its high sequence identity (>99%) to the new scuticociliate lineage. This ASV represented an important node in the community network of the reservoir hypolimnion and was closely associated with co-occurring prokaryote ASVs affiliated with typical hypolimnetic taxa, namely members of genera *Methylobacter* and *Rubinisphaera* (*Planctomycetaceae*) [15], and family *Nitrosomonadaceae*.

Ciliate communities in hypolimnetic layers experience more severe food limitations compared to epilimnetic ones, which select for ciliate species that are able to cope with these stable and adverse conditions. If environmental conditions are stable relative to species’ generation times, it promotes competitive exclusion whereas exogenous and endogenous fluctuations are considered as major factors promoting coexistence in plankton [104, 105]. This is also the reason for a general trend of the larger functional diversity and community dynamics of epilimnetic ciliate assemblages as a response to rapidly changing prey availability, e.g. by replacement of algal dominants forming only short-lived abundance peaks, persisting for a few days to weeks [31, 59, 106]. In contrast, the prominent scuticociliate species represents a very good example of a specialized filter-feeding bacterivorous species that likely meets all its carbon requirements by feeding solely on hypolimnetic prokaryotes available in moderate densities (Fig. 1B). Moreover, we never observed prey cells/particles larger than 2 μm in the scuticociliate food vacuoles (Fig. 2A-F).

The comparable rates of predator and prey growth rates detected under *in situ* temperature represent very rare examples of estimating the microbial dynamics in cold hypolimnetic waters. Besides the low temperature, the growth of the scuticociliate *in situ* should be regulated by the prey food quality and abundance related also to the distinct taxa of functional groups of hypolimnetic prokaryotes [15, 35, 36, 103, 107]. The strong impacts of the prey characteristics were also suggested by the data from experimental flow-through systems using the bacterivorous scuticociliate *Cyclidium glaucoma* [108].

Slightly smaller cells of a similar *Cyclidium*-like scuticociliate (MCV of 550 μm3), found in the reservoir epilimnion during summer at water temperatures around 20°C, showed ~2.3-fold higher uptake rates (470 prokaryotes h^−1^ cell^−1^) under prokaryotes’ densities of 3-4 million per ml [87]. Moreover, the latter study demonstrated higher clearance rates and strong size-selective grazing of the *Cyclidium*-like scuticociliate on picocyanobacteria of larger cells than the typical bacterioplankton cells. Besides, many typical hypolimnetic prokaryotes such as representatives of *Nitrosomonadaceae*, *Methylobacter*, *Planctomycetota*, and *Verrucomicrobiota* [15, 35, 36] possess relatively large cell volumes. Thus, preferential grazing on larger prokaryotic cells in the hypolimnion can result in higher growth rate of scuticociliates by gaining more biomass per cell ingested, because for ciliate growth estimates we used only the MCV of hypolimnetic prokaryotes, without considering the possible effect of the prey size selectivity.

### Concluding remarks

Building on both classical morphology-based and modern sequencing approaches, we significantly contributed to the limited knowledge of the role of different ciliates species and HNF communities as bacterivores under specific cold-water hypolimnetic conditions. Our study, through high-frequency sampling, also documents a remarkable stability and overall functional simplification of the hypolimnetic ciliate community with the dominance of one highly specialized and so far undescribed lineage of bacterivorous scuticociliates. Regardless of the low water temperature, this hypolimnetic scuticociliate showed substantial growth dynamics (doubling in 4–6 days) and high prokaryotic uptake rates. Moreover, our study suggests that insufficiently studied hypolimnetic protistan communities host important but as yet undescribed ciliates, but also other bacterivorous protistan species. They significantly contribute to the overall bacterioplankton mortality rates, nutrient recycling, and a temporal accumulation of inorganic nutrients during the stratification phase in these cold habitats.

## Supplementary Material

Simek_et_al_Revised_Supplementary_information_wraf148

## Data Availability

All data generated or analyzed during this study are included in this published article (and its supplementary information files). The sequence data generated from the 18S rRNA gene long amplicon sequencing was submitted to the European Nucleotide Archive (ENA) and is available under the BioProject: PRJEB84054.
