# Supplementary material for: New lineage of scuticociliates dominates the ciliate community and bacterivory in hypolimnetic waters of a freshwater reservoir
Source: ISME J. 2025 Jul 13;19(1):wraf148. doi: 10.1093/ismejo/wraf148 (PMC12366787; doi:10.1093/ismejo/wraf148)

**Supplementary Information**

**New lineage of scuticociliates dominates the ciliate community and bacterivory in hypolimnetic waters of a freshwater reservoir**

Karel Šimek^a^, Usman Asghar^a,b^, Bettina Sonntag^c^, Vojtěch Kasalický^a^, Tanja Shabarova^a^, Indranil Mukherjee^a^

^a^ Biology Centre CAS, Institute of Hydrobiology, Na Sádkách 7, 370 05 České Budějovice, Czech Republic

^b^ Faculty of Science, University of South Bohemia, 37005, České Budějovice, Czech Republic

^c^ Research Department for Limnology, Mondsee, University of Innsbruck, Mondseestrasse 9, A-5310 Mondsee, Austria

**This file includes:**

Supplementary Figures S1-S4

**Supplementary Figures**

**Figure S1.** Examples of three depth vertical profiles over the study period in the Římov reservoir (7^th^ and 16^th^ August and 6^th^ September, 2023): Water temperature, oxygen and Chl-*a* concentrations, and penetration of photosynthetically active radiation (PHAR). A dashed line shows the position of the sampled hypolimnetic layer of a 25 m depth.





**Figure S2.** Time-course changes in concentrations of total dissolved phosphorus (**A**), dissolved reactive phosphorus (**B**), dissolved nitrogen (**C**), and dissolved organic carbon (**D**) in the reservoir epilimnion and hypolimnion between 7^th^ August and 15^th^ September, 2023.

**

**

**Figure S3.** Time-course changes of water temperature and chlorophyll-*a* concentrations in the Římov reservoir epilimnion between 7^th^ August and 15^th^ September, 2023.





**Figure S4.** Microphotographs of the protargol stained hypolimnetic ciliates: (**A**) *Balanion* *planctonicum;* (**B**) *Cinetochilum* *margaritaceum*, arrow points to characteristic notch at the posterior; (**C**, **D**) *Coleps* *hirtus*, upper arrow denotes furrow between anterior and posterior main plates, posterior arrow denotes furrow between main and collateral plates; (**E**, **F**) *Cyrtolophosis* *mucicola;* (**G**) *Halteria* sp.; (**H**, **I**) *Mesodinium* *pulex;* (**J**) *Mesodinium* *acarus*; (**K**, **L**) *Rimostrombidium* *brachykinetum*, arrows denote anterior and posterior end of one ciliary row; (**M**) *Stokesia* *vernalis;* (**N**, **O**) *Vorticella* sp.; (**P**, **Q**, **R**, **S**) *Urotricha* *globosa*. AG – anterior girdle of ciliary tufts, AM – adoral organelles/membranelles, AP – anterior main plates, AT – apical ciliary tuft, AZM – adoral zone of membranelles, CC – caudal cilium, CR – ciliary rows, EX – extrusomes, JB – jumping bristles, MA – macronucleus, MI – micronucleus, MG – middle girdle of ciliary tufts, OA- oral apparatus, OB – oral basket, OF – oral flaps, PP – posterior main plates, PR – tentacular processes, ST – stalk, UM – undulating membrane. Scale bars 10 µm (E, F, J, K, L, P, Q, R, S), 20 µm (A, B, C, D, G, H, I, N, O), 100 µm (M).


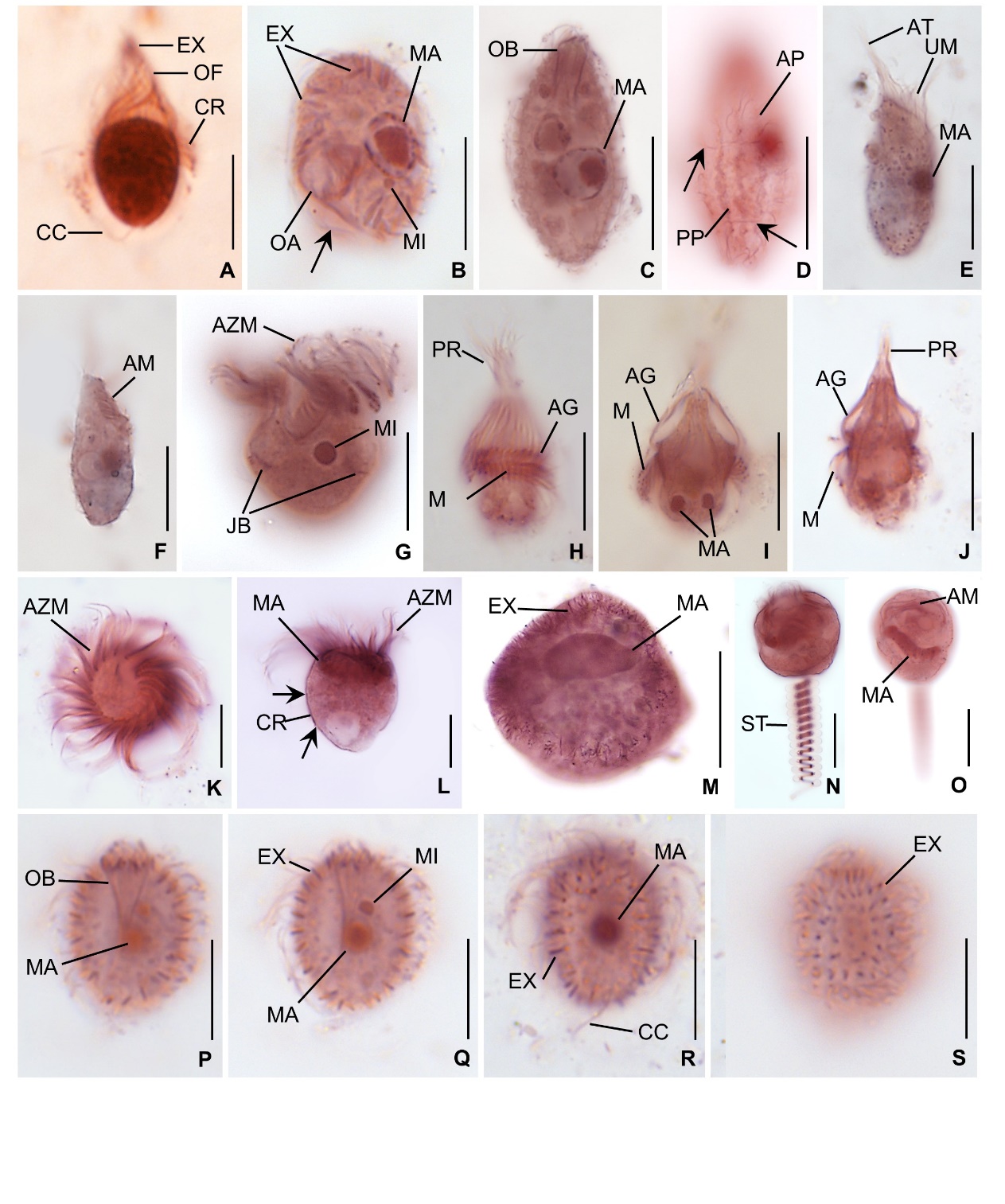

Supplement: Simek_et_al_Revised_Supplementary_information_wraf148 [file simek_et_al_revised_supplementary_information_wraf148.docx]
